# Impact of Ethnicity and Underlying Comorbidity on COVID-19 Inhospital Mortality: An Observational Study in Abu Dhabi, UAE

**DOI:** 10.1155/2021/6695707

**Published:** 2021-03-01

**Authors:** Asma Deeb, Khulood Khawaja, Nida Sakrani, Abdulla AlAkhras, Ahmed Al Mesabi, Ravi Trehan, Palat Chirakkara Kumar, Zahir Babiker, Nico Nagelkerke, Emmanuel Fru-Nsutebu

**Affiliations:** ^1^Division of Paediatric Endocrinology, Sheikh Shakhbout Medical City, Abu Dhabi, UAE; ^2^Division of Paediatric Rheumatology, Sheikh Shakhbout Medical City, Abu Dhabi, UAE; ^3^Internal Medicine Department, Sheikh Shakhbout Medical City, Abu Dhabi, UAE; ^4^Division of Infectious Diseases, Sheikh Shakhbout Medical City, Abu Dhabi, UAE; ^5^Division of Orthopaedic Surgery, Sheikh Shakhbout Medical City, Abu Dhabi, UAE; ^6^Division of Neurology, Sheikh Shakhbout Medical City, Abu Dhabi, UAE; ^7^Public Health Institute, UAE University, Al Ain, UAE

## Abstract

**Background:**

The UAE reported its first cluster of COVID 2019 in a group of returned travellers from Wuhan in January 2020. Various comorbidities are associated with worse disease prognosis. Understanding the impact of ethnicity on the disease outcome is an important public health issue but data from our region is lacking.

**Aim:**

We aim to identify comorbidities among patients hospitalized for COVID-19 that are associated with inhospital death. Also, to assess if ethnicity is correlated with increased risk of death. *Patients and Method*. The study is a single-centre, observational study in Shaikh Shakhbout Medical City, Abu Dhabi. Patients admitted with COVID-19, between 1^st^ of March and the end of May, were enrolled. Records were studied for demography, comorbidity, and ethnicity. Ethnicity was divided into Arabs (Gulf, North Africa, and the Levant), South Asia (India, Pakistan, Bangladesh, Nepal, and Afghanistan), Africans, the Philippines, and others. The study was approved by the Department of Health of Abu Dhabi.

**Results:**

1075 patients (972 males) were enrolled. There were 24 nationalities under 5 ethnicity groups. Mean (average) age was 51 years (20–81). 101 (9.4%) died with deceased patients being significantly older. Death risk was not significantly influenced by sex. Duration of hospitalization among survivors was 6.2 days (0.2–40.4) with older patients and men staying longer (*P* < 0.01). Comorbidities of diabetes, hypertension, cardiovascular disease, chronic renal disease, liver disease, and malignancy were associated with higher risk of mortality univariate, but only liver disease reached statistical significance after adjustment for age. The highest percentage of death was seen in Arab Levant (21.2) followed by the Asian Afghan (18.8); however, differences among ethnicities did not reach statistical significance (*P* = 0.086).

**Conclusion:**

COVID-19 outcome was worse in older people and those with comorbidities. Men and older patients required longer hospitalization. Ethnicity is not seen to impact the risk of mortality.

## 1. Introduction

Coronavirus disease 2019 (COVID-19) is caused by severe acute respiratory syndrome coronavirus 2 (SARS-CoV-2). The disease was initially identified as the cause of an outbreak of infection in Wuhan, China, at the end of December 2019. Since then, the virus has spread to most countries in the world and has been declared a pandemic [1]. The United Arab Emirates (UAE) reported its first cluster of COVID-19 cases in a group of returned travellers from Wuhan in late January 2020 [2]. UAE has since ramped up its testing and treatment capacity and applied a robust nationwide public health campaign to identify, isolate, and trace confirmed cases. A total of 166,502 cases have been confirmed in the UAE as of November, 2020, with a first surge of the disease witnessed between April and June [3].

The severity of the disease and its outcome have been variable. It is suggested that patients who develop severe illness and develop a more severe inflammatory response might have experienced longer virus exposure times [4]. In a meta-analysis by Hu, the risks of severity and mortality ranged from 12.6 to 23.5% and from 2.0 to 4.4%, respectively [5]. The estimated mortality was 1.1% in nonsevere patients and 32.5% in severe cases during the average 32 days of follow-up period. [6]. In a meta-analysis by Fu, the overall estimated proportion of severe cases and case-fatality rate was 25.6% and 3.6%, respectively [7]. Mortality for those requiring mechanical ventilation has been reported as 88.1% [8].

Various comorbidities were found to be associated with higher disease severity and worse outcome and death. A meta-analysis that included 76,993 patients with COVID-19 showed that most common comorbidities associated with poorer prognosis were diabetes mellitus, hypertension, cardiovascular diseases, smoking, chronic obstructive pulmonary disease, malignancy, and chronic kidney disease [9]. Similar results were reported by others [5, 8, 10, 11]. Notably, mortality rates were found to be considerably higher in patients with diabetes compared with nondiabetes patients [12]. This findings led to great concerns in the UAE which has one of the world's highest prevalence rate of diabetes [13].

Differences in mortality rates between people with different ethnicity background have been identified in multiple studies. Black populations and ethnic minority communities are found not only to be at an increased risk of infection with severe disease but also to have more frequent adverse outcomes, including death [14–16].

Understanding of the role of ethnicity and socioeconomic status in the risk of acquiring COVID-19 infection and outcome is important for health service planning, targeting prevention efforts, and future vaccine development. However, such data are limited. The UAE witnessed a significant population increase during the past few years because of major growth in the various economic sectors, which lead to an influx of workers from diverse cultural and religious background. The population of the UAE increased from 4.1 million in 2005 to roughly 9.7 million as of midyear 2019 [17]. An estimated 11.5% of the UAE population is made up of UAE citizens and the remaining 88.5% made up of Expatriate workers. The largest group of non-UAE nationals are South Asian 59.4% (includes Indians 38.2%, Bangladeshi 9.5%, Pakistani 9.4%, and others 2.3%), Egyptian 10.2%, Filipino 6.1%, and other 12.8% [18]. This wide cosmopolitan nature of the UAE enables the studying impact of disease in different ethnicities who share the same environment. Results of such studies are of paramount importance to establish public health measures.

## 2. Aim

We aim to identify comorbidities among patients hospitalized for COVID-19 that are associated with inhospital death. Also, to assess whether ethnicity is correlated with increased risk of death due to COVID-19 in the UAE.

## 3. Methods

The study is a single-centre, retrospective, observational study in Shaikh Shakhbout Medical City (SSMC), Abu Dhabi, UAE. Adult patients who were admitted to SSMC with confirmed COVID-19 during the period from 1^st^ of March to the end of May were included. Electronic medical records of the patients were reviewed. A two-pronged approach was used to ensure all patients were identified. These were through records tagged with the relevant international classification of diseases–10 (ICD–10) codes and laboratory records of confirmed cases. Records were studied for patients' age, sex, duration of hospitalization, and disease outcome (discharged or deceased). Nationalities were retrieved and ethnicity was recorded under different categories. The Arab ethnicity was subdivided into Arab Levant (Iraq, Lebanon, Jordan, Syria, and Palestine), North African Arabs (Egypt, Algerian, and Morocco), and Gulf Arabs (UAE, Oman, and Yemen). Other ethnicity categories were South East Asians (Afghan, Nepal, Pakistan, India, and Bangladesh), African (sub-Saharan), Philippines, and others. Comorbidities included obesity (Body mass index of more than 30 kg/m^2^), diabetes, hypertension, cardiovascular disease (ischemic heart disease and heart failure), chronic renal disease, chronic obstructive airways disease, chronic liver disease, and malignancy. The study was approved by the Central Department of Health Institution Research Board in Abu Dhabi and the SSMC local committee (MAFREC-192).

### 3.1. Statistical Method

Sample size was determined by the total number of eligible patients. Statistical analysis was carried out using standard univariate methods to compare groups, such as t-tests and chi-square tests, as well as stepwise (forward selection, LR method) logistic regression and linear regression for multivariate analyses of binary and continuous outcomes, respectively. SPSS v.22 was used for all analyses. A significance level of 0.05 was used throughout. The Mantel-Haenszel (log rank) test was used to compare “survival” times. Kaplan-Meier curves were used to graphically present hospitalization duration. For missing data on obesity for 35 surviving patients, estimated probability for being obese (based on their age and sex) was used as their obesity value (e.g., someone with a 20% probability of being obese received the “obesity value” 0.20).

## 4. Results

1075 adult patients were hospitalized during the study period. 972 were males and 103 females. 101 (9.4%) died and 974 got discharged. Mean (average) duration of hospitalization was 6.2 days (0.2–40.4). Overall mean (average) age was 51 years (20–81).

### 4.1. Duration of Hospitalization

**Figure 1:**
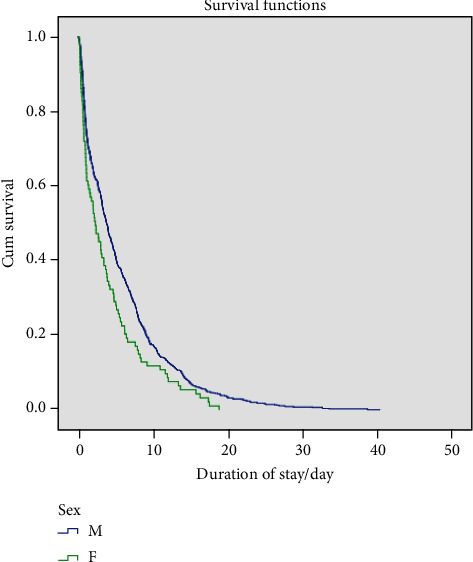
Kaplan-Meier curves of sex difference of hospitalisation duration among surviving patients.

Surviving patients were discharged after an average of 5.6 days (SD: 5.9), while deceased patients stayed on average 12.0 days (SD: 8.5) in hospital. Among surviving patients, the duration of hospitalisation depended on age and sex. A linear regression of duration of stay on age and sex showed that the duration increased by 0.058 (se .016, *P* < 0.01) days for each year of age and that men stayed on average 1.63 (se .64, *P* < 0.05) days longer than women. Duration of hospitalisation for females was both univariately (log rank test *P* = 0.004) and by linear regression, significantly shorter than those of males (Figure 1).

### 4.2. Age, Sex and Risk of Death

Out of the 972 male patients, 91 died while there were 10 female deaths out of 93 patients (*P* > 0.5). Deceased patients were older (54.7, SD13.3) than surviving patients (45.1, SD11.8). Odds ratio for death per year of age (by logistic regression, univariately) was estimated at 1.061(95% CI 1.044–1.079).

### 4.3. Comorbidities and Risk of Death

187 patients were obese, of whom 29 died, while 72 nonobese patients died out of 694. 284 patients had diabetes. Of those, 50 died. 41 patients of 214 who had hypertension died. 69 patients had cardiovascular disease of whom 18 died. 12 patients (out of 37) with chronic kidney disease died and 6 out of 12 with malignancy died (Table 1). Comorbidities of diabetes, hypertension, cardiovascular disease, chronic renal disease, liver disease, and malignancy were univariately significantly associated with a higher risk of mortality. We further undertook stepwise logistic regression of death on age, sex, obesity, and various comorbidities to identify independent risk factors for mortality. Age (OR per year = 1.061, 95% CI 1.044-1.079, *P* < 0.0001) and chronic liver disease (OR = 8.99 9% CI: 1.18-66.22, *P* = 0.034) were found to be significant predictors of death.

### 4.4. Ethnicity

The cohort had 24 nationalities which were categorized under 5 different ethnicity groups. 172 patients were Arabs (Gulf 102, North Africa 34, and Levant 33). 871 were South Asians (Pakistani 290, Indian 247, Bangladeshi 202, Nepalese 46, and Afghan 16). 37 patients were Africans and 33 from the Philippines. 14 patients were collected from Europe, China, New-Zealand, and Indonesia. Odds ratio for overall risk of death with 95% confidence interval was 1.620 (0.884–2.968). To examine the statistical significance of ethnicity on risk of death, stepwise logistic regression was done with death as an outcome using a 2-sided chi-square test. While the highest percentage of death was seen in Arab Levant (21.2%) followed by the Asian Afghan (18.8), the difference did not reach statistical significance (*P* = 0.086) (Table 2).

## 5. Discussion

Global evidence shows a greater COVID-19 burden on people with specific characteristics, among those are older age, male sex, obesity, comorbidities, and poverty [19–21]. There has been overwhelming data on the impact of age as an adverse outcome of the disease. Older patients had more likelihood of a severe disease requiring intensive care admission and had a higher case fatality rate [7, 22]. A study by Grasselli et al. described 1591 patients referred for intensive care treatment in Milan, Italy. The majority were older men who required mechanical ventilation and had a mortality rate of 26% [23]. A multivariate analysis concluded that older patients, male gender, comorbidities, and time from disease onset to hospitalization were all significantly associated with death [24]. Our results are in concordance with these international results. We found that deceased patients were older (54.7, SD13.3) than surviving patients (45.1, SD11.8). The mean age of deceased patients in our cohort is lower than that reported internationally but this might reflect the relatively young population of the UAE.

Impact of sex on COVID-19 has been extensively studied. A meta-analysis included 38 studies with 3062 COVID-19 patients showed that a higher proportion of infected patients were male in whom the incidence rate of respiratory failure was 19.5% and the fatality rate was 5.5%. [25]. In our study, we had a higher number of male patients presenting with the disease and requiring admission. However, we did not find a statistically significant difference between male and female mortality. Of note, our study looked at the hospitalized patients only, and while this observation applies to our cohort, it might not necessarily have implication for gender differences in the infection fatality rate in the UAE. Furthermore, the higher number of male patients can be explained by the high numbers of male migrant workers in the UAE.

Studies showed an average duration of hospitalization for COVID-19 of 12 ± 4 days [8, 10, 26] with longer duration of hospitalization for critically sick patients and those who died [27]. We found that deceased patient required a longer admission duration compared to survivors with a linear relationship of increased duration for each year of age. It was interesting to see that men required a longer duration of hospitalization than women with a statistical significance of *P* < 0.05 (Figure 1).

Various comorbidities have been identified as predisposing factors for disease severity and death. A meta-analysis by Emami included 76.993 patients with COVID-19 showed that the most common comorbidities associated with poor prognosis were hypertension, cardiovascular diseases, diabetes mellitus, chronic obstructive pulmonary disease, malignancy, and chronic kidney disease [9]. Similar results were reported by others [5, 8, 10, 11]. In particular, males with comorbidities presented a higher risk of being critically ill than males without comorbidities [28]. In a large cohort of 1035 COVID-19 patients, it was found advanced age and an increasing number of comorbidities are independent predictors of inhospital mortality for COVID-19 patients [27]. Diabetes, in particular, was implicated to be a significant factor related to prognosis. Higher rates of admission to intensive care and mortality were seen in patients with diabetes [12]. We found a statistically significant difference in death in people who have diabetes among our study cohort, but this disappeared after adjustment for age which is strongly associated with the prevalence of diabetes. Besides diabetes, obesity is also noted to be associated with severe disease requiring intensive care admission and mechanical ventilation [8, 29]. We did not find obesity a significant contributory factor to death in our cohort (Table 1). However, this could be related to our moderate sample size rather than being a genuine result. Cardiovascular disease and hypertension were seen to be predictors for severe disease particularly if they were seen in older patients [30, 31]. We found a significant difference in risk of death in patients with underlying hypertension, cardiovascular disease, renal disease, liver disease, and malignancy, but some of these associations seem to have been, in part, confounded by age except for liver disease which is shown to be a predictor of death regardless of age (Table 1). However, as the number of patients with liver disease was small, this finding needs independent confirmation.

Ethnicity and socioeconomic status influence health outcomes particularly for infectious diseases. Experience from previous pandemics has shown that ethnic minorities, and those with lower socioeconomic classes have been disproportionately affected [32]. Evidence from the current COVID-19 pandemic suggests the same trend, but confirmatory research remains limited [21]. There are 1518 COVID-19 studies registered on ClinicalTrials.gov. However, only six are collected data on ethnicity [15]. Risk of infection, severe disease, and death are reported to be higher in black and ethnic minority populations [14, 15, 33]. Data showed that 33% of patients critically ill with confirmed COVID-19 in intensive care in the UK were from black and ethnic minority groups, despite these comprising only about 13% of the UK population [34]. Similarly, black people in the USA represent 13·4% of the population. Yet, they account for between 28% and 70·5% of deaths with risk of infection that is about three times higher than in predominantly white communities [16]. Similar data suggested that COVID-19 has disproportionately sickened Hispanic communities in the United States [35]. A prospective cohort study using the UK biobank showed that socioeconomic deprivation and low education level were consistently associated with a higher risk of COVID infection. In addition, the study concluded that some minority ethnic groups have a higher risk of infection regardless of the socioeconomic conditions [36]. In this study, the black and South Asian groups appeared to be at greatest risk, with Pakistani ethnicity at greatest risk within the South Asian group. Our study showed the highest percentage of death in Arab Levant (21.2%) followed by the Asian Afghan (18.8); however, the difference among different ethnicities did not reach statistical significance (*P* = 0.086) (Table 2). Considering that the COVID-19 pandemic has placed multiple extraordinary stressors on migrant workers, we hypothesized to see a higher percentage of adverse outcome in South Asian patients who constitute a high proportion of labour workforce in the UAE. However, this hypothesis was not proven. This could be due to the fact that our study is undertaken in a single centre and that we only looked at hospitalized people who sought medical care. Our study design would not enable identification of heterogeneities in the risk of infection. Along with these lines, a cumulative risk assessment framework for migrant workers in Kuwait was developed in recognition of the vulnerability of this population in acquiring infections [37]. Socioeconomic disadvantage is linked to living in overcrowded housing. Similarly, some ethnic groups like Bangladeshi, Indian, and Chinese are more likely to live in extended families with multiple generations [38]. This lifestyle is hypothesized to increase risk of infection transmission [39]. Living in extended families and mixing of large generation family members at mealtimes is heavily practiced in the UAE. It is a strongly associated with cultural beliefs of family unit rather than socioeconomic status.

Our study has limitations as it is undertaken in a single centre and is retrospective. Also, it lacks detailed socio-economic status and living circumstances. However, it adds knowledge to the sparse data available from the region on the COVID-19 characteristics. It is undertaken in Abu Dhabi/UAE where the population is a mixture of different ethnicities. Collecting data about ethnicity is a major issue as status is not always documented with the health care data. The settings of patients' demographics in our centre employ self-reporting of ethnicity and country of origin which is known to be the gold standard approach in such studies [40].

We conclude that COVID-19 inhospital mortality is higher in older people and those with comorbidities. Men and older patients required longer hospitalization. We could not confirm that ethnicity impacts the risk of mortality among those hospitalized for the disease.

## Figures and Tables

**Table 1:** Comorbidities in surviving and deceased patients. Percentage indicated in relation to total group within individual comorbidity.

Comorbidity	Y/N (number in subcategory)	Survived (% within subcategory)	Died (%)	Univariate *P* value
Obesity	N (766)	694 (90)	72 (9.4)	0.11
	Y (216)	187 (87)	29 (13)	
	Unknown (93)	93	0	

Diabetes	N (741)	690 (93)	51 (7)	<0.001
	Y (334)	284 (85)	50 (15)	

Hypertension	N (820)	760 (93)	60 (7)	<0.001
	Y (255)	214 (84)	41(16)	

Cardiovascular disease	N (988)	905 (92)	83 (8)	<0.01
	Y (87)	69 (79)	18 (21)	

COPD	N (1073)	972 (91)	101 (9)	>0.5
	Y (2)	2 (100)	0 (0)	

Asthma	N (1045)	946 (91)	99 (9)	>0.5
	Y (30)	28 (93)	2 (7)	

Chronic renal disease	N (1026)	937 (91)	89 (9)	<0.01
	Y (49)	37 (76)	12 (24)	

Liver disease	N (1071)	972 (91)	99 (9)	<0.0
	Y (4)	2 (50)	2 (50)	

Malignancy	N(1057)	962 (91)	95 (9)	<0.01
	Y (18)	12 (67)	6 (33)	

**Table 2:** Death rate based on ethnicity with percentages in relation to the subgroup.

	Total	Death	Recovered	% of death within subgroup
Arab all	172	26	146	15.1
Arab Levant	33	7	26	21.2
Arab Gulf	102	14	88	13.7
Arab North Africa	34	2	32	5.9
South Asia all	871	140	731	16.1
Asian Afghan	16	3	13	18.8
Asian Nepalese	46	5	41	10.9
Asian Indian	247	22	225	8.9
Asian Bangladeshi	202	17	185	8.4
Asian Pakistani	290	23	267	7.9
African	37	2	35	5.4
Filipino	23	4	19	17.4
Others	14	2	12	14.3

## Data Availability

Data is available if required.
